# Calcification and growth rate recovery of the reef-building *Pocillopora* species in the northeast tropical Pacific following an ENSO disturbance

**DOI:** 10.7717/peerj.3191

**Published:** 2017-04-11

**Authors:** Jose de Jesús A. Tortolero-Langarica, Alma P. Rodríguez-Troncoso, Amílcar L. Cupul-Magaña, Juan P. Carricart-Ganivet

**Affiliations:** 1 Laboratorio de Zoología Marina, Tecnológico Nacional de México, Instituto Tecnológico de Bahía de Banderas, Bahía de Banderas, Nayarit, México; 2 Laboratorio de Ecología Marina, Centro de Investigaciones Costeras, Centro Universitario de la Costa, Universidad de Guadalajara, Puerto Vallarta, Jalisco, México; 3 Unidad Académica de Sistemas Arrecifales, Instituto de Ciencias del Mar y Limnología, Universidad Nacional Autónoma de México, México

**Keywords:** Coral density, Thermal stress, Extension rate, Annual calcification, Central Mexican Pacific

## Abstract

Pocilloporids are one of the major reef-building corals in the eastern tropical Pacific (ETP) and also the most affected by thermal stress events, mainly those associated with El Niño/Southern Oscillation (ENSO) periods. To date, coral growth parameters have been poorly reported in *Pocillopora* species in the northeastern region of the tropical Pacific. Monthly and annual growth rates of the three most abundant morphospecies (*P. cf. verrucosa*, *P. cf. capitata*, and *P. cf. damicornis*) were evaluated during two annual periods at a site on the Pacific coast of Mexico. The first annual period, 2010–2011 was considered a strong ENSO/La Niña period with cool sea surface temperatures, then followed by a non-ENSO period in 2012–2013. The linear extension rate, skeletal density, and calcification rate averaged (±SD) were 2.31 ± 0.11 cm yr^−1^, 1.65 ± 0.18 g cm^−3^, 5.03 ± 0.84 g cm^−2^ yr^-1^ respectively, during the strong ENSO event. In contrast, the respective non-ENSO values were 3.50 ± 0.64 cm yr^−1^, 1.70 ± 0.18 g cm^−3^, and 6.02 ± 1.36 g cm^−2^ yr^−1^. This corresponds to 52% and 20% faster linear extension and calcification rates, respectively, during non-ENSO period. The evidence suggests that *Pocillopora* branching species responded positively with faster growth rates following thermal anomalies, which allow them to maintain coral communities in the region.

## Introduction

Coral growth is the key factor to building reef with calcareous hard structures that provide direct and indirect habitats for marine species and consequently maintain the growth (accretion) and structure of coral reef ecosystems (Hubbard, Miller & Scaturo, 1990; Guzmán & Cortés, 1993; Sheppard, Davy & Pilling, 2010). Despite the importance of coral growth, in the last decades calcification rate have been declining due to different threats including anthropogenic and natural factors (Kleypas, McManus & Menez, 1999; Carilli et al., 2009; Cantin & Lough, 2014). In order to recognize these effects in the continuous coral decline, different parameters such as linear extension rate (cm yr^−1^), skeletal density (g cm^−3^) and calcification rate (g cm^−2^ yr^−1^) have been helpful to assess coral growth rate over time (Lough & Cooper, 2011). This allows us to know how the organisms are responding to regional environmental factors, which thereby allows for the modeling of future accretion of the coral reef on an ecosystem level (Knutson, Buddemeier & Smith, 1972; Lough & Barnes, 2000; Lough & Cooper, 2011).

Coral physiological processes, such as calcification, are regulated by exogenous factors such as light, temperature, hydraulic energy, sedimentation and depth (Lough & Barnes, 2000; Lough & Cooper, 2011). Coral growth is also regulated by endogenous factors such as size, age, reproduction cycles and different endosymbiont types in the coral colony (Sheppard, Davy & Pilling, 2010; Lough & Cooper, 2011; Allemand et al., 2011). It is known that temperature may control coral growth and calcification rates (Lough & Barnes, 2000; Lough & Cooper, 2011). For example, the increase in the sea surface temperature (SST) does not exceed the species thermal threshold, it can benefit coral development (Sheppard, Davy & Pilling, 2010). However, studies have also shown that extreme increases in temperature can be detrimental (Hoegh-Guldberg, 1999). Anomalous SST, characterized by temperatures more than 1 °C above the maximum (in summer) or below the minimum (in winter) monthly mean, may stress the coral-symbiont resulting in coral bleaching (Hoegh-Guldberg, 1999; Hoegh-Guldberg & Fine, 2004). Thermal stress and bleaching can intensify during two phases of the El Niño/Southern Oscillation (ENSO) phenomena (Hoegh-Guldberg, 1999; Wang & Fiedler, 2006). The warm anomaly phase (>30.5 °C) and La Niña, the cold anomaly phase (<22 °C) (Wang & Fiedler, 2006; Hoegh-Guldberg & Fine, 2004; Cupul-Magaña & Calderón-Aguilera, 2008). Consequently, both El Niño and La Niña periods may decrease the amount of metabolic energy needed for coral growth, posing a direct threat to their survival (Furla et al., 2005; Wang & Fiedler, 2006).

Recently, ENSO events occurring with greater intensity and frequency producing an impact over coral reefs worldwide (Hoegh-Guldberg, 1999; Timmermann et al., 1999; Glynn, 2000; Hoegh-Guldberg et al., 2007; Lee & McPhaden, 2010). Historically, records show the most severe events in the eastern tropical Pacific (ETP) resulted in massive coral bleaching and high mortality rates. The warm phase El Niño, with uncharacteristically high temperatures, caused average loss of 74% (ranging from 50–97%) of coverage during 1982–1983 and an average 39% (ranging from 3–98%) in 1997–1998 (Glynn, 1990; Timmermann et al., 1999; Glynn, 2000; Lee & McPhaden, 2010). The cold phase of La Niña episodes also has affected coral population and survivorship in the region (Glynn, Veron & Wellington, 1996; Carriquiry et al., 2001; Reyes-Bonilla et al., 2002; Cupul-Magaña & Calderón-Aguilera, 2008; Paz-García, Balart & García-de-Léon, 2012; Glynn et al., 2017). In spite of these periodic losses, a glimmer of hope has been observed in several coral reefs where there have been encouraging signs of recovery after the negative impacts of ENSO events in the ETP (Guzmán & Cortés, 2007; Glynn et al., 2015).

In the ETP, branching corals such as *Pocillopora* species show high calcification and extension growth rates in contrast to the massive and sub-massive corals *Pavona* and *Porites* species (Guzmán & Cortés, 1989; Guzmán & Cortés, 1993; Manzello, 2010; Norzagaray-López et al., 2014). However, upwelling and non-upwelling zones, inshore and offshore reefs, depth and temperature gradients including the susceptibility to the effects of ENSO (El Niño and La Niña) events may produce difference on *Pocillopora* growth along the region (Glynn, 1977; Glynn, Veron & Wellington, 1996; Guzmán & Cortés, 1989; Jiménez & Cortés, 2003; Dullo, 2005; Manzello, 2010; Paz-García, Balart & García-de-Léon, 2012).

In the last decade, studies have reported a decline of 11–21% on calcification rates in the tropical zones of the Great Barrier Reef in Australia (Cooper et al., 2008; Tanzil et al., 2013; De’ath, Fabricius & Lough, 2013). In the equatorial zone of the ETP, a model of coral growth described by Manzello (2010) predicted a constant reduction (0.9% yr^−1^) of skeletal extension under the effects of increased acidification (Hoegh-Guldberg et al., 2007). However, according to their life history and acclimatization process, each species develops a particular growth response regulated by local and regional environmental conditions and it is probable that, at different latitudes, corals may acclimatize differently (Jiménez & Cortés, 2003; Hoeke et al., 2011; Glynn et al., 2015). At this point, it is expected that each species associated to a specific area will acclimatize differently and produce variability in parameters such as growth and reproduction (Jiménez & Cortés, 2003; Hoeke et al., 2011). Therefore, more studies are vital to distinguish the variability of growth patterns of reef building corals throughout the Pacific.

The objectives of this study were: (1) to compare annual extension rate, skeletal density, and calcification rate among three *Pocillopora* morphospecies at two annual periods (ENSO/La Niña and non-ENSO) (2) to compare bimonthly extension rate and SST, and (3) to contrast data from this study with other studies of annual extension rate, throughout the ETP. The results provide information on the coral response during and after thermal stress anomalies and may shed light on their ability to cope with future scenarios of ENSO events.

## Materials and Methods

### Study area

**Figure 1:**
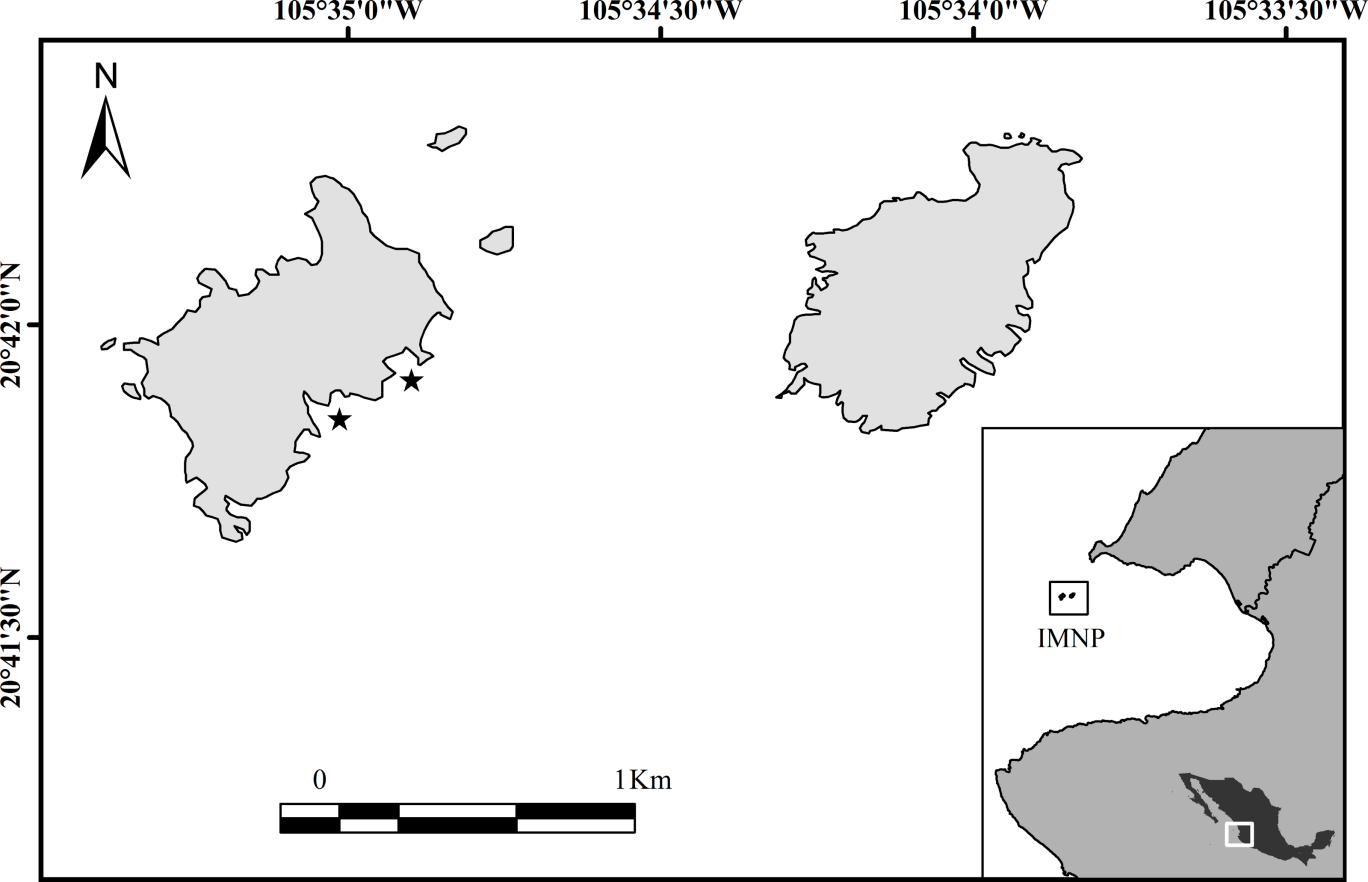
Study location, Islas Marietas National Park, Mexico (IMNP) at the northeast tropical Pacific (NETP). The stars indicate where growth studies were implemented.

The Islas Marietas National Park (IMNP) is a group of islands (0.76 km^−2^) located 7 km offshore of the Mexican Pacific coast in the northeast tropical Pacific (Fig. 1). They are volcanic and surrounded by rocky and fringing coral reefs between 1 to 18 m deep (CONANP, 2007). The coral community is dominated by *Pocillopora* spp. at the shallower depths (1–9 m) and *Porites* spp. and *Pavona* spp. at mid-shallow and deeper depths (5–18 m). The study location is situated in an oceanographic transition area, influenced by three intra-annual currents: California Current (CC) carrying cold waters and low salinity (34.6) from the North (18−21 °C), evident from January to March (Kessler, 2006; Pennington et al., 2006; Pantoja et al., 2012); Mexican Coastal Current (MCC) providing warm waters from the South (27−30 °C) during July to November; and the Gulf of California Current (GCC) carrying warm waters with high salinity (>34.9), present from September to October (Kessler, 2006; Pennington et al., 2006; Palacios-Hernández et al., 2010; Pantoja et al., 2012).

### Field and laboratory processing

The study was conducted during a two year period from December 2010 to December 2011 and from February 2012 to February 2013. The sampling was performed with the support of the authorities of the National Park and the permission No. DGOPA.04552.040711.1798. During both annual periods of the study, were sampled coral fragments resulted by the fragmentation caused by water energy and bio-erosion by different organism (natural fragmentation). These fragments were collected at 3–5 m depth from several meters apart between each other’s. All coral samples were classified at morphospecies level based on skeletal morphology using taxonomic descriptions (Veron, 2000; Schmidt-Roach et al., 2014). In order to distinguish the different types of morphospecies in this study we referred as “*Pocillopora* cf. specie”.

### First growth period

During the first period 2010–2011 (Strong ENSO/La Niña), 15 colonies for each of three *Pocillopora* morphospecies (*P.* cf. *verrucosa n* = 15, *P.* cf. *damicornis n* = 15 and *P.* cf. *capitata n* = 15) were collected and immediately transported to the laboratory (Universidad de Guadalajara), in coolers filled with local seawater. In the laboratory coral fragments were evenly distributed in four aquaria, each having filters (Elite^®^), heaters (Titanium Via Aqua^®^), full spectrum lights (Halminton^®^) and previously equipped with coastal seawater from near the site of collection. Fragments were acclimated at 25.5 °C (temperature registered during the sampling) for 24 h to reduce the stress received by handling. In order to generate a reference line of initial growth, organisms were stained using red Alizarin Sigma^®^ (15 mg 1^−1^) for 24 h (Barnes, 1970), and no evidence of dead tissue was recorded during the staining process. After the stain, coral fragments were re-located in clean seawater aquaria and monitored for 48 h noting bleaching or tissue damage. Corals were then returned and placed on stable substrate (coral rubble) using plastic cable ties. Coral extension was measured *in situ* every 2-months (cm mo^−1^) during the 2010–2011 period; briefly, calipers (0.05 mm precision) were used to record maximum apical height measured from the bottom to the top of each fragment. After one year of monitoring, the coral fragments of the first period were collected (December 2011) for further analysis.

### Second growth period

**Figure 2:**
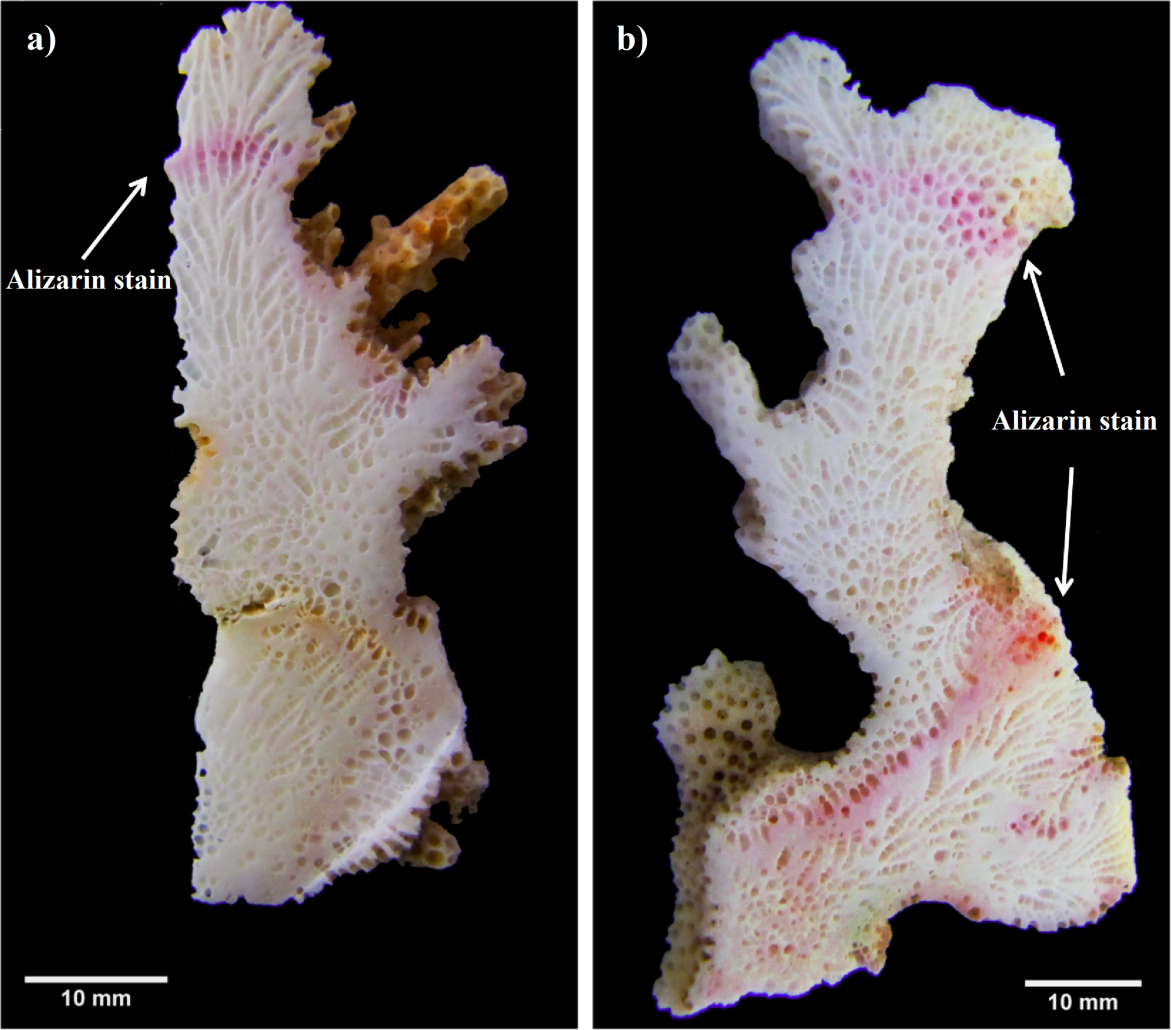
*Pocillopora* morphospecies coral fragment staining method. (A) Using single stained method to quantify only one annual period. (B) Using double stained method in order to reference the beginning and end of the second annual period.

Another similar growth study was performed in 2012–2013 (non-ENSO). A total of 48 fragments of three *Pocillopora* morphospecies. (*P.* cf. *verrucosa* [*n* = 25], *P.* cf. *damicornis* [*n* = 8] and *P.* cf. *capitata* [*n* = 15]) were collected (January 2012) and stained as previously described. Monthly coral extension measurements were made bimonthly (cm mo^−1^) using the same method as for the first experiment period (2010–2011). In order to generate a final growth mark line, colonies of the second experiment period (2012–2013) were re-stained *in situ* one year later (February 2013) by covering each colony with a plastic bag containing Alizarin red (15 mg 1^−1^) for four hours (Fig. 2).

### Annual growth parameters

After one year of growth (in both periods), *Pocillopora* fragments develop branching colonies with tridimensional complex growth forming many new branches around the colony. Annual growth parameters were analyzed in a 27 sample subset of *Pocillopora* colonies ([*n* = 9], three per morphospecies for 2010–2011 and [*n* = 12] *P.* cf. *verrucosa,* [*n* = 3] *P.* cf. *damicornis* and [*n* = 3] *P.* cf*. capitata* for 2012–2013), that were free of bleaching and disease and absent of signs of bioerosion. In order to eliminate any organic matter, all colonies were air dried and exposed to sun light for 48 h placed in a convection oven at 70 °C for 5 h. Branches of each colony were sectioned and separated proximally, but below the initial Alizarin stain line using a diamond-tipped saw blade (Qep^®^). The resulting coral slices were used to obtain annual growth data. Annual extension rate (cm yr^−1^) was measured using digital calipers (Mitutoyo^®^, 0.001 mm precision) by noting the maximum linear distance between the initial stain line and the tip of the new skeletal growth for corals of the 2010–2011 period and from the first to the second stain line for those corals of the 2012–2013 period (Gladfelter, 1984; Manzello, 2010). This procedure was achieved using 4–6 sub-samples (branches) for each colony. Density data in this study were referred to as skeletal density, which was obtained from the sub-samples using the buoyant weighing method (Gladfelter, 1984; Bucher, Harriott & Roberts, 1998). Colony density was calculated as the mean mass (dry weight) divided by the mean of the volume of water displaced (wet weight) based on data obtained of each branch examined (4–6 per colony). Calcification rates were calculated as the product of the mean annual linear extension rate and the mean skeletal density of each colony and expressed as g cm^−2^ yr^−1^ (Dodge & Brass, 1984; Chalker, Barnes & Isdale, 1985; Carricart-Ganivet & Barnes, 2007). Temperature data were recorded during the whole experiment at 25 min intervals using thermographs (HOBO pendant) that were installed at the study site.

### Data analysis

Mean ± standard deviation (SD) of growth parameters were obtained at genus and species level using colony-level data for each species. Parametric and non-parametric analysis of variance (ANOVA and Kruskal–Wallis) were performed in this study. For example, during the first period only skeletal density data fit to parametric assumptions for this reason we used ANOVA test. For other hand extension and calcification rate does not fit parametric assumptions, in this case we evaluate differences between morphospecies using Kruskal-Wallis (non-parametric). Relationships between calcification rate, extension rate, and density were tested using linear regression. Growth parameter differences between the two annual periods were evaluated using a General Linear model two-way ANOVA with fixed effects of species, time period, and their interaction. Tukey’s HSD test were employed to analyzed multiple comparisons and determine significant differences, *p-values* of <0.05 were adjusted by a factor of six (Species × year) in order to obtain final critical *p-values* (Bonferroni correction). Daily temperature data was pooled in monthly and annual average (±SD) from December 2010 to February 2013. The difference of SST between the two annual periods was assessed month by month using *t*-test. Additionally, information of SST was pooled in bimonthly average in order to correlate using a simple linear regression, with bimonthly growth for each period. Afterwards, in response to temperature anomalies (La Niña and El Niño), a model of coral growth (cm yr^−1^) was constructed by using non-linear regression (Peak, Gaussian, 4 Parameter) of the historical annual extension rate literature in the ETP, including data of this study (Table 1) and the SST anomalies based on the Oceanic Niño index 3.4 (http://ggweather.com/enso/oni.htm). All statistical analyses were evaluated with a minimum confidence interval of 95% (α = 0.05) using Sigma Plot Ver. 11 (SPSS) and Statistical Ver. 8 (Stats) software.

**Table 1:** Mean growth parameters (95% confidence) of *Pocillopora* morphospecies reported in literature for the eastern tropical Pacific and the ENSO category (intensity) associated for each period, based on the Oceanic Niño Index 3.4 (ONI).

				Growth rate			
Species	Locality	Period	ENSO	Density	Extension	Calcification	Reference
			intensity	(g cm^−3^)	(cm yr^−1^)	(g cm^−2^ yr^−1^)	
*Pocillopora* cf. *damicornis*	Golfo de Panamá, Panamá	1971–1974	Strong Niño/Niña	–	3.08 (2.55–3.61)	–	Glynn (1977)
	Golfo de Chiriquí Panamá	1972–1974	Strong Niño/Niña	–	3.86 (3.39–4.33)	–	Glynn (1977)
	Onslow, Galápagos	1975	Strong Niña	–	2.24	–	Glynn, Wellington & Birkeland (1979)
	Caño Island, Costa Rica	1985-1987	Strong Niño	–	3.46 (2.13–4.37)	–	Guzmán & Cortés (1989)
	Golfo de Chiriquí Panamá	1989–1990	Non-ENSO	–	3.32 (3.17–3.47)	–	Eakin (1996)
	Clipperton Atoll	1988-1993	–	–	2.54 (1.85–3.45)	–	Glynn, Veron & Wellington (1996)
	Golfo de Papagayo Costa Rica	1996–1997	Non-ENSO	–	4.55 (2.80–6.10)	–	Guzmán & Cortés (1993)
	Golfo de Chiriquí Panamá	2003–2004	Non-ENSO	2.05 (1.92–2.17)	2.82 (2.44–3.12)	5.75 (5.27–6.65)	Manzello (2010)
	Golfo de Chiriquí Panamá	2005–2006	Weak Niña	2.01 (1.83–2.24)	2.75 (2.40–3.30)	5.50 (4.92–5.92)	Manzello (2010)
	Southern Pacific of México	2010–2013	Strong Niña/Non-ENSO	1.78	2.94	5.23	Medellín-Maldonado et al. (2016)
	Central Pacific of México	2010–2011	Strong Niña	2.22 (2.09–2.36)	2.31 (2.14–2.47)	5.62 (5.29–5.96)	Present study
	Central Pacific of México	2012–2013	Non-ENSO	1.68 (1.36–1.99)	3.73 (3.21–4.25)	5.99 (4.87–7.11)	Present study
*Pocillopora* cf. *verrucosa*	Southern Pacific of México	2010-2013	Strong Niña/Non-ENSO	1.47	3.42	5.04	Medellín-Maldonado et al. (2016)
	Central Pacific of México	2010–2011	Strong Niña	2.15 (1.97–2.34)	2.69 (2.50–2.89)	5.82 (5.66–6.05)	Present study
	Central Pacific of México	2012–2013	Non-ENSO	1.66 (1.52–1.79)	3.69 (3.29–4.09)	6.10 (5.37–6.82)	Present study
*Pocillopora* cf. *capitata*	Southern Pacific of México	2010-2013	Strong Niña/Non-ENSO	1.67	2.92	4.87	Medellín-Maldonado et al. (2016)
	Central Pacific of México	2010–2011	Strong Niña	2.08 (1.94–2.21)	2.31 (2.01–2.69)	4.81(4.50–5.12)	Present study
	Central Pacific of México	2012–2013	Non-ENSO	1.44 (1.14–1.73)	3.93 (3.35–4.50)	5.31 (4.23–6.39)	Present study

## Results

### First growth period (2010–2011)

**Figure 3:**
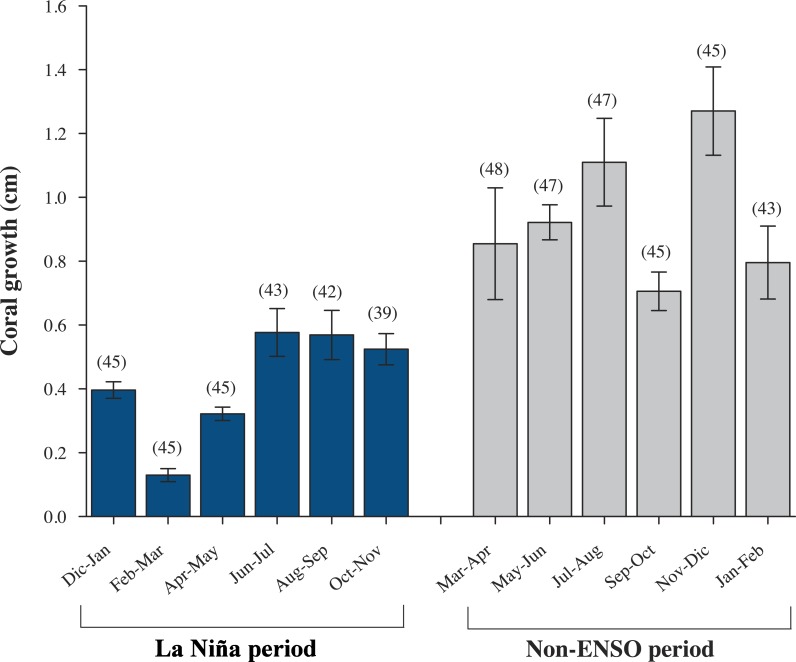
Monthly growth rates (±SD) of *Pocillopora* morphospecies in the northeast tropical Pacific. Bars represent, bimonthly growth (cm mo^−1^); blue, during a strong La Niña (2010–2011) period, and gray, Non-ENSO (2012–2013)period. Parenthesis represent number of coral colonies.

**Figure 4:**
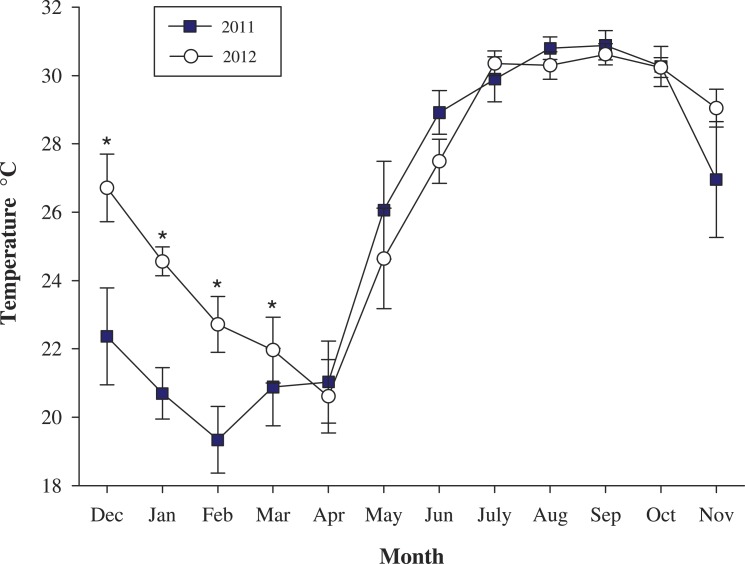
Mean monthly (±SD) sea surface temperatures (SST). Comparisons of La Niña (ENSO) and normal SST period. La Niña period, shaded squares and Non-ENSO period, open plots. ^∗^ Statistical differences, *P* =  < 0.001.

Data from all nine colonies: *P.* cf. *verrucosa* (*n* = 3), *P.* cf. *capitata* (*n* = 3), *P.* cf. *damicornis* (*n* = 3), show a mean (±SD) linear extension of 2.31 ± 0.11 cm yr^−1^, skeletal density of 1.65 ± 0.18 g cm^−3^ and calcification rate of 5.03 ± 0.84 g cm^−2^ yr^−1^ (Table 1). However, there was no significant difference on annual linear extension rate (Kruskal-Wallis; *H*_2,6_ = 0.958, *P* = 0.619), skeletal density (ANOVA; *F*_2,6_ = 4.276, *P* = 0.070), nor calcification rates (Kruskal-Wallis; *H*_2,6_ = 1.263, *P* = 0.532) across the three morphospecies. The relationship between linear extension and calcification rates ( *r*^2^ = 0.523, *P* = 0.028; Fig. S1A), and skeletal density with calcification (*r*^2^ = 0.556, *P* = 0.021; Fig. S1B) was positive; however, there was no significant relation of linear extension and skeletal density (*r*^2^ = 0.104, *P* = 0.397; Fig. S1C). At genus level *Pocillopora* species exhibited a bimonthly growth increase ranging between 0.15–0.64 cm mo^−1^ (Fig. 3), where the colder months February–March showed the lowest growth rates (<0.15 cm mo^−1^) and the minimum SST 18.3 °C (Fig. 4 and Table S1).

### Second growth period (2012–2013)

From a total of eighteen colonies: *P.* cf. *verrucosa* (*n* = 12), *P.* cf. *capitata* (*n* = 3), *P.* cf. *damicornis* (*n* = 3), a total of 80 *Pocillopora* branches with an average of four per colony (ranging 4–6), were analyzed resulting in a mean linear extension growth of 3.50 ± 0.64 cm yr^−1^, skeletal density of 1.70 ± 0.18 g cm^−3^ and calcification rate of 6.02 ± 1.36 g cm^−2^ yr^−1^ at genera level. Once again, *P.* cf. *verrucosa*, *P.* cf. *capitata* and *P.* cf. *damicornis* did not show any differences on linear extension (ANOVA; *F*_2,15_ = 0.066, *P* = 0.936), skeletal density (Kruskal-Wallis; *H*_2,15_ = 3.868, *P* = 0.145) nor calcification rates (ANOVA; *F*_2,15_ = 0.730, *P* = 0.498) (Table 1). There was a positive relationship between linear extension and calcification (*r*^2^ = 0.781, *P* =  < 0.001; Fig. S1D), and skeletal density with calcification (*r*^2^ = 0.396, *P* = 0.012; Fig. S1E), whereas the linear extension and skeletal density was not related (*r*^2^ = 0.0388, *P* = 0.481; Fig. S1F). At genus level *Pocillopora* morphospecies exhibited a bimonthly growth increase ranging between 0.71–1.27 cm mo^−1^. Lowest growth rates, <0.95 cm mo^−1^ (Fig. 3) were observed during the warmest months ∼31 °C (September–October) (Fig. 4 and Table S1).

### Thermal anomalies and coral growth

The temperature data show that mean sea surface temperature (SST) from December 2010 to November 2011 was 26.58 ± 4.44 °C. The minimum was recorded between February–March (17.5 °C) and the maximum during August–September (32.2 °C). The abnormal mean monthly values of SST (≤2°C) were associated with a strong “La Niña” in the early winter of 2011 (Fig. 4) (Lee & McPhaden, 2010). During the second period (February 2012–2013), mean SST was 26.36 ± 4.04 °C with the lowest temperatures registered between March to April (19.8 °C) and the highest between September to October (31.8 °C). Data showed statistical differences in winter temperatures associated to the period December–March between 2011 and 2012, (*df* = 6, *t* =  − 2.580, *P* = 0.04) (Fig. 4).

**Figure 5:**
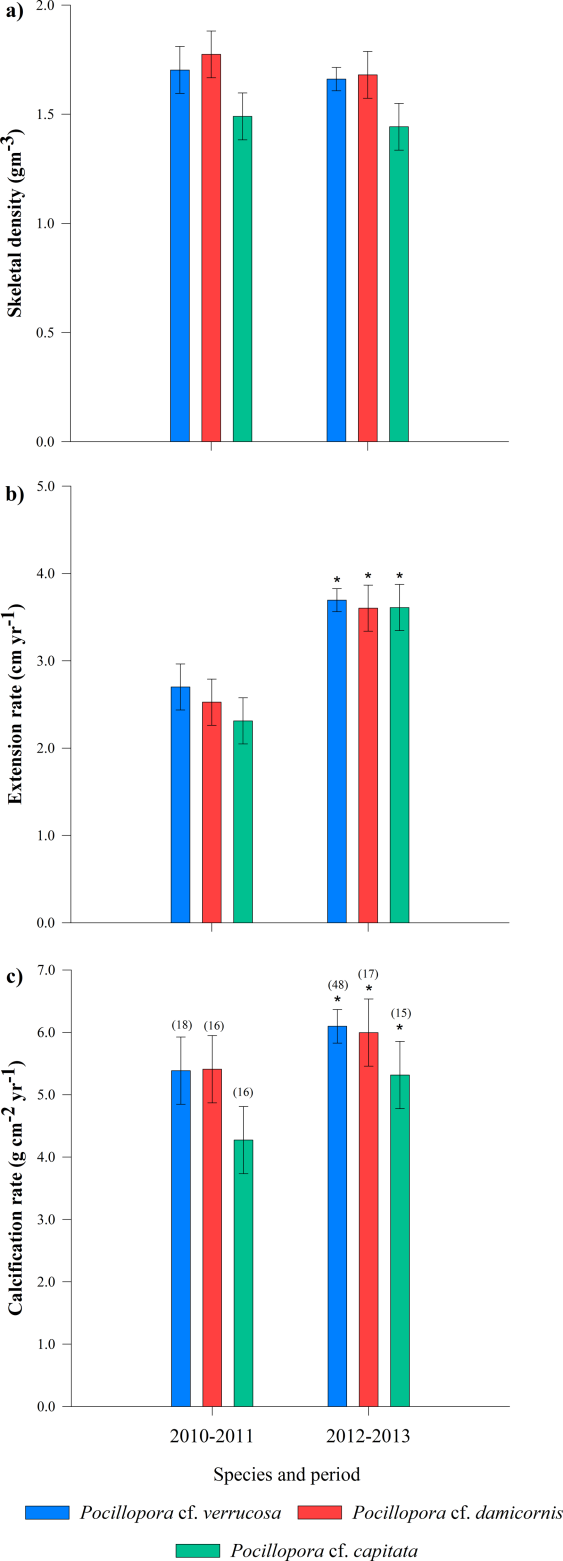
Annual growth parameters (±SD) of three *Pocillopora* morphosspecies during the two experimental periods 2010–2011 and 2012–2013. Comparisons between species from different periods. (A) skeletal density, (B) extension rate, and (C) calcification rate. Colors bars represent annual growth and the interaction of each species with the year. ^∗^ Statistical differences using Bonferroni correction. Parentheses represent number of coral branches used for all three parameters.

**Figure 6:**
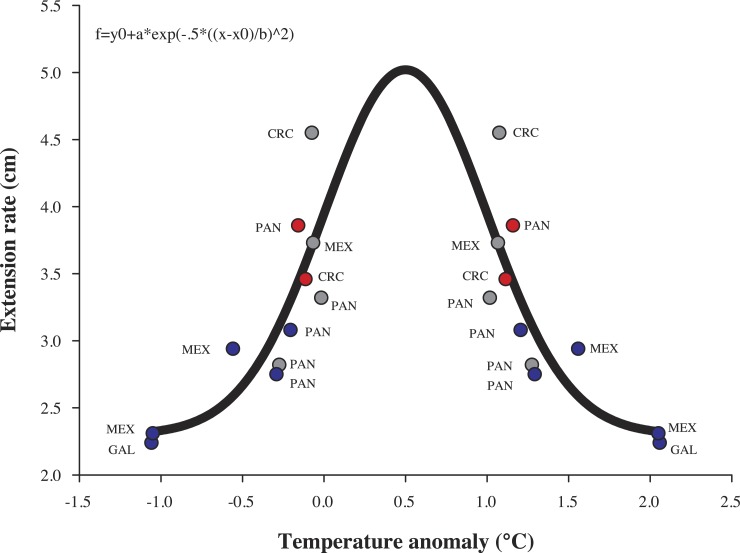
Model of coral growth of *Pocillopora* cf. *damicornis*. Relationship of historical coral growth (cm yr^−1^) with theoretical annual temperature anomalies that were influenced by ENSO in the eastern tropical Pacific. CRC, Costa Rica; PAN, Panamá; MEX, México; GAL, Galápagos. Blue dots, La Niña; Red dots, El Niño and Gray dots, Non-ENSO.

Annual growth differed significant by year in terms of extension rate and calcification rate (Table 2). However, growth data from both years, was no significant interaction (species × year, *P* > 0.05, Table 2) emerged in any metric. Tukey pairwise comparisons reflect differences between the years (La Niña vs Non-ENSO) within each morphospecies; *P.* cf. *verrucosa* (*P* = 0.003), *P.* cf. *damicornis* (*P* = 0.009) and *P.* cf. *capitata* (*P* = 0.002) (Fig. 5). Annual linear extension and calcification rate were 19.7% and 51.5% higher during 2012–2013 compared with the 2010–2011 period. Monthly growth rates (cm mo^−1^) exhibited a two times higher rate during the 2012–2013 period subsequent to La Niña event (Fig. 3). The data of historical coral growth with the ENSO intensity associated to each period was fitted to a Gaussian peak (*r* = 0.832, *P* = 0.001; Fig. 6).

**Table 2:** The results of two-way ANOVA’s of *Pocillopora* morphospecies in three coral growth parameters and the interaction of the species and the year.

Parameter	Source	DF	MS	*F*	*P*
Extension rate	Species	2	0.107	0.510	0.608
	Year	1	6.489	30.992	<0.001^*^
	Species × Year	2	0.043	0.206	0.816
Skeletal density	Species	2	0.121	3.516	0.048
	Year	1	0.019	0.555	0.465
	Species × Year	2	0.001	0.039	0.961
Calcification rate	Species	2	1.887	2.166	0.140
	Year	1	5.162	5.785	0.024
	Species × Year	2	0.085	0.098	0.907

**Notes.**

* Represent significant differences using Bonferroni correction.

## Discussion

Growth parameters evaluated in this study, suggest that *Pocillopora* corals from the northeast tropical Pacific are increasing their capacity to tolerance frequent thermal anomalies, which is quick recovery followed by growth activity. Nevertheless, ENSO events are affecting the coral communities with more severity, intensity and frequency (Timmermann et al., 1999; Hoegh-Guldberg et al., 2007; Lee & McPhaden, 2010), there is no certainty that these coral species will respond similarly in the long-term. Continual coral growth monitoring is indispensable to understand the change of coral growth over time. The comprehensive monitoring of linear extension rate, skeletal density and calcification rates will help generate information to enhance comprehensive strategies of conservation on coral reef ecosystems.

We indicate here that coral growth in the northeast tropical Pacific showed similar values compared with other areas from ETP (Table 1), thus suggesting the acclimatization ability to inhabit different conditions. This feature may confer resilience to local thermal anomalies (Harrison, 2011; Paz-García et al., 2015). Although differences between *Pocillopora* morphospecies were not observed (Fig. 5), they are four times faster in calcification rates compared with other predominant genera of *Pavona* and *Porites* (Guzmán & Cortés, 1989; Cabral-Tena et al., 2013; Medellín-Maldonado et al., 2016). This large difference corroborates the importance of the *Pocillopora* genera in the region, being as the major producer of calcium carbonate and one of the most dominant reef building species of the ETP region (Glynn & Wellington, 1983; Guzmán & Cortés, 1989; Carriquiry et al., 2001; Norzagaray-López et al., 2014; Medellín-Maldonado et al., 2016).

### Growth parameters and the ENSO influence

The *Pocillopora* morphospecies in this study had similar rates of linear extension compared to published records across 40 years in the ETP (Table 1). A regional difference within the ETP is observed between the equatorial zone (3°S–10°N) and the northern zone (15°N–29°N) of the ETP; where the equatorial zone shows a continuous decline of 0.9% year^−1^ on linear extension rate (Manzello, 2010) compared to a non-apparent change in the northern zone (Table 1). However, the populations we recorded north of the ETP show annual growth variations with higher extension (52%) and calcification rates (20%) following a cool ENSO period than during it (Table 1). These were also reflected on monthly growth rates, where they showed a growth recovery following La Niña episode (Fig. 3 and Table S1). The results support that abnormal SST caused by ENSO/La Niña produces a negative effect observed on coral growth (Fig. 6), given differences in growth between annual periods measured in the same area (Fig. 5 and Table 1). Despite the importance of ENSO in coral growth, their effects have been taken lightly into account in linear models of growth, and consequently may produce misperception in the annual trend of coral growth across the time (Anderson, Heron & Pratchett, 2014). Therefore, coral extension growth (cm yr^−1^) is also locally regulated by the local environmental conditions such as light irradiance, temperature, local upwelling, nutrient load, aragonite saturation and pH (Glynn, 1977; Jiménez & Cortés, 2003; Wellington & Glynn, 1983; Guzmán & Cortés, 1989; Hughes, 1987; Dullo, 2005; Manzello, 2010; Manzello, Eakin & Glynn, 2017), which in turn are influenced by anomalous events (ENSO) in several locations of the ETP (Table 1). The synergy of environmental factors may affect growth parameters over the life history of *Pocillipora* species due to the high sensibility to abrupt changes in temperature, light irradiance, hydraulic energy and pH (Paz-García, Balart & García-de-Léon, 2012; Lough & Cooper, 2011; Glynn et al., 2017).

The modulation of growth was also reflected on skeletal density with slightly lower values during 2012–2013 compared with 2010–2011 when temperatures were relatively cold (Fig. 4). Cool temperatures may favor denser skeletal deposition rather than extension (Fig. 5). This resulted in an inverse pattern in comparison with annual periods that there was non-ENSO (SST anomalies) present (Allemand et al., 2004; Carricart-Ganivet, 2007). Therefore, the differences of annual growth may be associated to ENSO events, where decreases on growth during 2010–2011 period allows for space competition with other groups, including the increased activity by bio-eroding species during the process of recovery-repair time (Glynn, 1985; Eakin, 1996). However, when the thermal stress ends, the corals are allowed to grow and a decrease on erosion and competition is observed (Eakin, 1996; Cortés & Guzmán, 1998; Hoegh-Guldberg, 1999; Jiménez et al., 2001; Jiménez & Cortés, 2003). At this point, results show that *Pocillopora* corals have acclimatized to high thermal range, by saving their energy to repair and to recover from thermal anomalies during the period of ENSO events (Guzmán & Cortés, 2007 ; Glynn et al., 2015). This is supported by the relationship between growth parameters, where calcification rate resulted positively with both extension and density (Fig. S1). Thereby, skeletal extension and density are regulated according to the demands of the environment, which allows successful survivorship.

In this study using similar methods, calcification rate of *Pocillopora* morphospecies showed similar rates compared with localities of Panamá during La Niña periods but a faster recovery rate (14%) after ENSO episodes (Table 1) (Manzello, 2010). Therefore, localities from the same region respond differently on calcification rate, this showing that corals may acclimatize differently to environmental variation. The ability to cope and recover from the stress events may be associated with the life history (Gates & Edmunds, 1999; Middlebrook, Hoegh-Guldberg & Leggat, 2008) and their specific symbiosis with the microalgae *Symbiodinium* type D, which is thermo-tolerant to high temperatures (LaJeunesse et al., 2010). Corals harbor *Symbiodinium* type D to ensure their organic osmolite providers maintain enough energy budget to resist abnormal conditions. Also, they continue investing in growth even under thermal stress conditions, which results in a positive acclimatization process to the organism (Glynn et al., 2001; Rodríguez-Troncoso, Carpizo-Ituarte & Cupul-Magaña, 2010; Brown & Cossins, 2011; Cunning et al., 2014). Due to these features of rapid acclimatization and growth recovery, *Pocillopora* species have been proposed as a potential tool for restoration of degraded coral reef (Tortolero-Langarica, Cupul-Magaña & Rodríguez-Troncoso, 2014).

The difference of annual calcification rate between subtropical (North) and tropical zone (Equatorial) in the eastern Pacific (Table 1), shows a similar pattern with corals from the same zones in the West Pacific, where coral growth has increased 4% in subtropical zones and is attributed to the slow increase (0.4 °C) of the SST in the last century (Lough & Barnes, 1997; Shi et al., 2012; Cooper, O’Leary & Lough, 2012; Lough, 2012). This is also confirmed, with the opposite effect reported on coral communities located in the tropical zone, where coral growth has decreased 11–21% associated with the steady increment of the SST (Cooper et al., 2008; De’ath, Lough & Fabricius, 2009; Tanzil et al., 2013; Ridd, Da Silva & Stieglitz, 2013). The SST keeps the organism on the limit of their thermal threshold and consequently decrease or inhibit the coral growth. It is evident that increase of SST caused by ocean warming in subtropical zones is beneficial to coral growth in the short-term, compared with lower rates in tropical zones. These results are consistent with the theory of slow shift of reef distribution to higher latitudes which may promote small changes on growth behavior in the near future (Poloczanska et al., 2013; Hoegh-Guldberg, 2014). This approach is actually a continuing idea, but does not support the notion of long-term resilience of coral reef ecosystems worldwide.

##  Supplemental Information

10.7717/peerj.3191/supp-1 Figure S1 Correlation of linear extension and skeletal density

10.7717/peerj.3191/supp-2 Table S1 Growth parameters of *Pocillopora* Mean monthly growth of three *Pocillopora* species in the Northeast Tropical Pacific, during the influence of La Niña and Neutral period.

10.7717/peerj.3191/supp-3 Data S1 Dataset of *Pocillopora* Dataset of growth parameters along the studied years with the description per specie.

## Additional Information and Declarations

### Field Study Permissions

The following information was supplied relating to field study approvals (i.e., approving body and any reference numbers): The sampling was performed with the support of the authorities of the National Park and the permission No. DGOPA.04552.040711.1798.

### Data Availability

The following information was supplied regarding data availability: The raw data has been supplied as a Supplementary File.
