# Supplementary material for: Calcification and growth rate recovery of the reef-building Pocillopora species in the northeast tropical Pacific following an ENSO disturbance
Source: PeerJ. 2017 Apr 11;5:e3191. doi: 10.7717/peerj.3191 (PMC5390766; doi:10.7717/peerj.3191)
Supplement: Table S1 — Mean monthly growth of three Pocillopora species in the Northeast Tropical Pacific, during the influence of La Niña and Neutral period. [file peerj-05-3191-s002.docx]

**Table S1.** Mean monthly growth of three *Pocillopora* morphospecies in the Northeast Tropical Pacific, during the influence of La Niña and Neutral period.

|  |  | **ENSO** | **Growth rate (cm mo^-2^) ± SD** | | | |
| --- | --- | --- | --- | --- | --- | --- |
| **Months** | **Period** | **Phase** | **Colonies**  **(n)** | ***Pocillopora* cf. *damicornis*** | ***Pocillopora* cf. *capitata*** | ***Pocillopora*  cf. *verrucosa*** |
| December-January | 2010-2011 | La Niña | 45 | 0.35 ± 0.03 | 0.38 ± 0.03 | 0.45 ± 0.03 |
| February-March | 2010-2011 | La Niña | 45 | 0.09 ± 0.02 | 0.15 ± 0.03 | 0.15 ± 0.03 |
| April-May | 2010-2011 | La Niña | 45 | 0.33 ± 0.02 | 0.34 ± 0.02 | 0.30 ± 0.02 |
| June-July | 2010-2011 | La Niña | 43 | 0.55 ± 0.02 | 0.55 ± 0.02 | 0.64 ± 0.03 |
| August-September | 2010-2011 | La Niña | 42 | 0.51 ± 0.02 | 0.60 ± 0.02 | 0.61 ± 0.04 |
| October-November | 2010-2011 | La Niña | 39 | 0.48 ± 0.02 | 0.52 ± 0.03 | 0.56 ± 0.03 |
|  |  |  |  | ***Pocillopora* cf. *damicornis*** | ***Pocillopora* cf. *capitata*** | ***Pocillopora*  cf. *verrucosa*** |
| March-April | 2012-2013 | Neutral | 48 | 0.91 ± 0.17 | 0.66 ± 0.17 | 0.95 ± 0.16 |
| May-June | 2012-2013 | Neutral | 47 | 0.78 ± 0.14 | 0.89 ± 0.13 | 0.79 ± 0.12 |
| July-August | 2012-2013 | Neutral | 47 | 1.23 ± 0.14 | 0.98 ± 0.13 | 0.90 ± 0.12 |
| September-October | 2012-2013 | Neutral | 45 | 0.95 ± 0.14 | 0.71 ± 0.13 | 0.60 ± 0.12 |
| November-December | 2012-2013 | Neutral | 45 | 1.17 ± 0.14 | 1.14 ± 0.13 | 0.75 ± 0.12 |
| January-February | 2012-2013 | Neutral | 43 | 0.97 ± 0.14 | 0.77 ± 0.13 | 0.66 ± 0.12 |
|  |  |  |  |  |  |  |
